# Intracellular Calcium Changes Correlate with Mitochondrial Dynamics After Differential Modulation of KATP Channels in a Cellular Model of Parkinson’s Disease

**DOI:** 10.1007/s11064-025-04598-2

**Published:** 2025-11-03

**Authors:** Andrea Evinova, Ivan Okruhlica, Peter Racay, Jan Strnadel, Erika Halasova, Renata Pecova, Michal Pokusa

**Affiliations:** 1 Jessenius Faculty of Medicine, Biomedical Centre Martin, Comenius University, Bratislava, Slovakia; 2 Department of Pathological Physiology, Jessenius Faculty of Medicine, Comenius University, Bratislava, Slovakia; 3 Department of Medical Biochemistry, Jessenius Faculty of Medicine, Comenius University, Bratislava, Slovakia

**Keywords:** KATP, Mitochondrial dynamics, Cell viability, Glibenclamide, Pinacidil, Diazoxide, 5-Hydroxydecanoate, Neurodegeneration

## Abstract

Up-to-date data on roles of ATP‑sensitive potassium (KATP) channels indicate their emerging roles in neurodegeneration. The aim of present study was to evaluate the significance of KATP channels on cell viability, calcium dynamics, and mitochondrial morphology with the accent on their intracellular localization. We distinguished between whole-cell KATP effects and specific effects of mitochondrial KATP under both physiological conditions and pathological conditions simulating in vitro Parkinson´s-type neurodegeneration. SH‑SY5Y cells with its high fidelity to dopaminergic neurons were treated for 24 h with the non‑selective KATP opener pinacidil and blocker glibenclamide, or with the mitochondrial KATP opener diazoxide and blocker 5‑hydroxydecanoate (5HD). The effects of modulators were analysed alone or alongside with rotenone, which is widely used as an inducer of Parkinson´s-type neurodegeneration. Intracellular calcium distribution and mitochondrial rebuild pattern was evaluated using the cell segmentation performed by fluorescent confocal microscopy. Although none of the KATP modulators reversed the negative effects of rotenone, significant and selective effects of mitochondrial KATP modulation on calcium homeostasis and mitochondrial morphology were observed. For antagonists, both compounds showed consistent effects, with non-selective glibenclamide exerting stronger effects, particularly in elevating calcium. More distinctive results were obtained for agonists: both reduced calcium concentration; however, pinacidil tended to induce mitochondrial fragmentation, an effect absent in diazoxide-treated cells. Furthermore, strong correlations were identified between calcium levels and several mitochondrial and cell viability parameters.

## Introduction

Although ATP-sensitive potassium (KATP) channel modulators were originally developed for the treatment of type 2 diabetes mellitus and certain cardiovascular diseases, their potential extends beyond these applications. After nearly three decades of research, KATP channels remain a significant focus in the field of neurodegeneration [1], due to their involvement in regulating cell death through effects on mitochondrial physiology, where these channels are also found [2]. Many experimental studies have demonstrated robust effects of KATP on the efficiency of mitochondrial respiration, dynamics, and calcium homeostasis [3]. Parkinson’s disease models are among the most commonly used systems to investigate KATP function channels in neurodegenerative pathogenesis [4] and some KATP modulators have already been confirmed as effective in these contexts [5].

In standard nomenclature, the key genes encoding KATP channel subunits are *KCNJ9* and *KCNJ11*, which produce the Kir6.1 and Kir6.2 subunits, respectively. These subunits assemble into a hetero/homotetrameric structures forming the potassium-permeable pore of the mature transmembrane channel [6, 7]. Although these channels are expressed in various tissues, their subunit composition varies significantly. Kir6.2 is predominantly expressed in neurons, while Kir6.1 is more prevalent in glial cells [8]. A similar distribution trend can be observed intracellularly, where Kir6.2 is also detected – though more weakly – on mitochondrial membranes [9]. In 2019, a novel transmembrane potassium channel encoded by *CCDC51* gene was identified [10]. Its structure resembles the canonical KATP channels, including a tetrameric ion-conducting pore. Although its expression appears pleiotropic across various human cell types, its intracellular localization has so far been confirmed exclusively at the mitochondrial level [11].

While all these channels mediate potassium flux across membranes, their functional diversity is shaped by their coupling with regulatory sulfonylurea receptors (SURs), which serve as ATP sensors. The SURs differ based on the pore-forming subunit: Kir6.2 typically associates with SUR1 or SUR2A [12], Kir6.1 with SUR2B [13], and CCDC51 with ABCB8 [10]. The specific SUR-subunit combinations, and their differential expression across tissues and intracellular compartments, likely underlie the conflicting outcomes observed in experimental studies using KATP modulators.

These modulators are commonly classified based on their affinity for specific SURs or pore-forming subunits. Glibenclamide (antagonist) and pinacidil (agonist) are generally considered as non-selective KATP modulators that affect channels throughout various cellular compartments [14, 15]. In contrast, diazoxide (agonist) and 5-hydroxydecanoate (antagonist) are thought to more selectively target mitochondrial KATP channels, with weaker affinity for those located in the endoplasmic reticulum or plasma membrane [16].

The major physiological consequence of KATP channel activity is regulation of calcium homeostasis, which is tightly linked to membrane potential modulation. In general, KATP channel inhibition leads to increased intracellular calcium levels [17], while activation has the opposite effect [18]. Numerous studies have also demonstrated the influence of KATP activity on mitochondrial network morphology, which is critical for maintaining mitochondrial efficiency through a balance of biogenesis and mitophagy [19–22]. Calcium homeostasis within mitochondria is also essential, as moderate calcium levels enhance tricarboxylic acid cycle (TCA) cycle activity, while excessive calcium accumulation may trigger apoptotic pathways [23].

Despite extensive experimental data, relatively little attention has been given to how the intracellular localization of KATP channels influences their specific effects. The present study aims to provide a detailed understanding of how selective activation or inhibition of KATP channels at different subcellular sites affects calcium dynamics and how these calcium fluctuations relate to changes in mitochondrial network morphology. The experimental approach was designed to distinguish the effects of mitochondrial-specific KATP activity from those of whole-cell KATP activity, under both physiological and pathological conditions. To distinguish possible beneficial KATP effects in condition of neurodegeneration, we employed SH-SY5Y cells, a dopaminergic progenitor cell line, under both physiological conditions and a Parkinson’s disease-like phenotype induced by complex I inhibition with rotenone.

## Materials and Methods

### Cell Culture Preparation

Human neuroblastoma SH-SY5Y cells (American Type Culture Collection, ATCC) were used as a dopaminergic neuronal model. Cells were cultured under standard conditions at 37 °C in a humidified atmosphere containing 5% CO₂. A non-differentiated phenotype was maintained using DMEM: F12 medium (Dulbecco’s Modified Eagle’s Medium and Ham’s F-12 Nutrient Mixture, Sigma-Aldrich) supplemented with 10% fetal bovine serum (FBS) and 1% penicillin-streptomycin solution (both from PAA). For experimental treatments, cells were exposed for 24 h to one of the following K_ATP channel modulators: 20 µM glibenclamide, 20 µM diazoxide, 10 µM pinacidil, or 250 µM 5-hydroxydecanoate (all from Merck). Each treatment was applied either alone or simultaneously with 50 nM rotenone (Sigma-Aldrich) for 24 h to induce mitochondrial stress.

### MTT Assay

SH-SY5Y cells were seeded at a density of 10,000 cells per well in 96-well plates. On the following day, cells were treated with KATP channel modulators and/or 50 nM rotenone for 24 h. Control cells received an equivalent volume of DMSO as a vehicle. After 20 h of treatment, 10 µl of MTT solution (5 mg/ml in PBS; Sigma-Aldrich) was added to each well, and cells were incubated for an additional 4 h at 37 °C. Subsequently, the resulting insoluble formazan crystals, formed by mitochondrial reduction of MTT (3-(4,5-Dimethylthiazol-2-yl)-2,5-Diphenyltetrazolium Bromide) in viable cells, were solubilized by adding 100 µl of SDS solution (0.1 g/ml) to each well, followed by overnight incubation at room temperature. The absorbance of dissolved formazan was measured at 570 nm using a Synergy H4 microplate reader (BioTek). Cell viability was expressed as a percentage of untreated control cells, whose absorbance values were defined as 100%. Each experimental condition was tested in four independent biological replicates.

### Confocal Microscopy

Mitochondrial morphology and basal cytosolic calcium levels were evaluated using a Zeiss LSM 880 laser scanning confocal microscope (LSM AxioExaminer platform) equipped with a W Plan-Apochromat 40×/1.0 DIC M27 water-immersion objective. SH-SY5Y cells were stained with MitoTracker Red TM (581/644 nm), ER-Tracker Blue-White DPX (~ 374 nm / 430–640 nm), and Fluo-4 AM calcium indicator (494/506 nm) (all from Thermo Fisher Scientific), following the manufacturer’s protocols. Imaging was conducted under identical acquisition conditions across all experimental groups. Five independent biological replicates were included per condition, with a minimum of five high-resolution fields of view acquired from sample in each replicate. Strength of lasers and master gain values were adjusted before measuring each biological replicate, and selected condition was used for recording whole images per distinct replicate.

### Colocalization Analysis

**Fig. 1:**
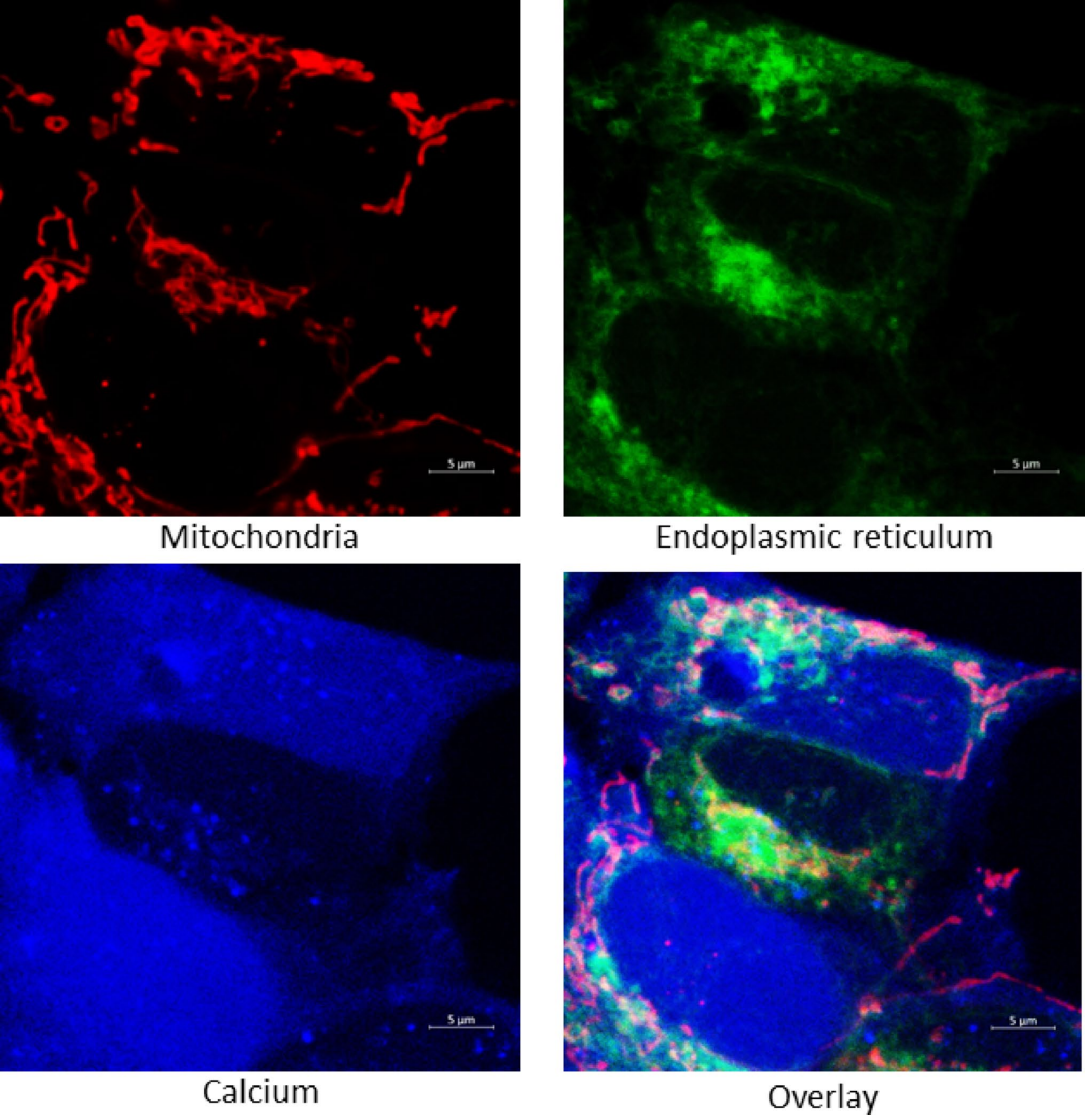
Overview of representative fluorescence microscopy images. Microscopy images showing calcium (Fluo-4 AM) fluorescence, mitochondrial (MitoTracker Red FM) and ER (ER-Tracker Blue-White DPX) signals. Fluo-4 AM fluorescence was used to assess calcium distribution in the whole cell and in colocalization with specific markers for mitochondria (and endoplasmic reticulum. Scale bars = 5 μm

To evaluate the pattern of colocalization between the Fluo-4 signal and markers of the mitochondrial network (MitoTracker Red FM) and endoplasmic reticulum (ER-Tracker Blue-White DPX), the colocalization module of ZEN Black software (Zeiss) was used. A representative image illustrating the colocalization of the respective signals is shown in Fig. 1. Background-corrected fluorescence intensities were calculated to estimate the relative calcium concentration indicated by the Fluo-4 probe in regions corresponding to mitochondrial and ER localization. For threshold setting, negative controls for each staining were used.

### Mitochondrial Network Analysis

**Fig. 2:**
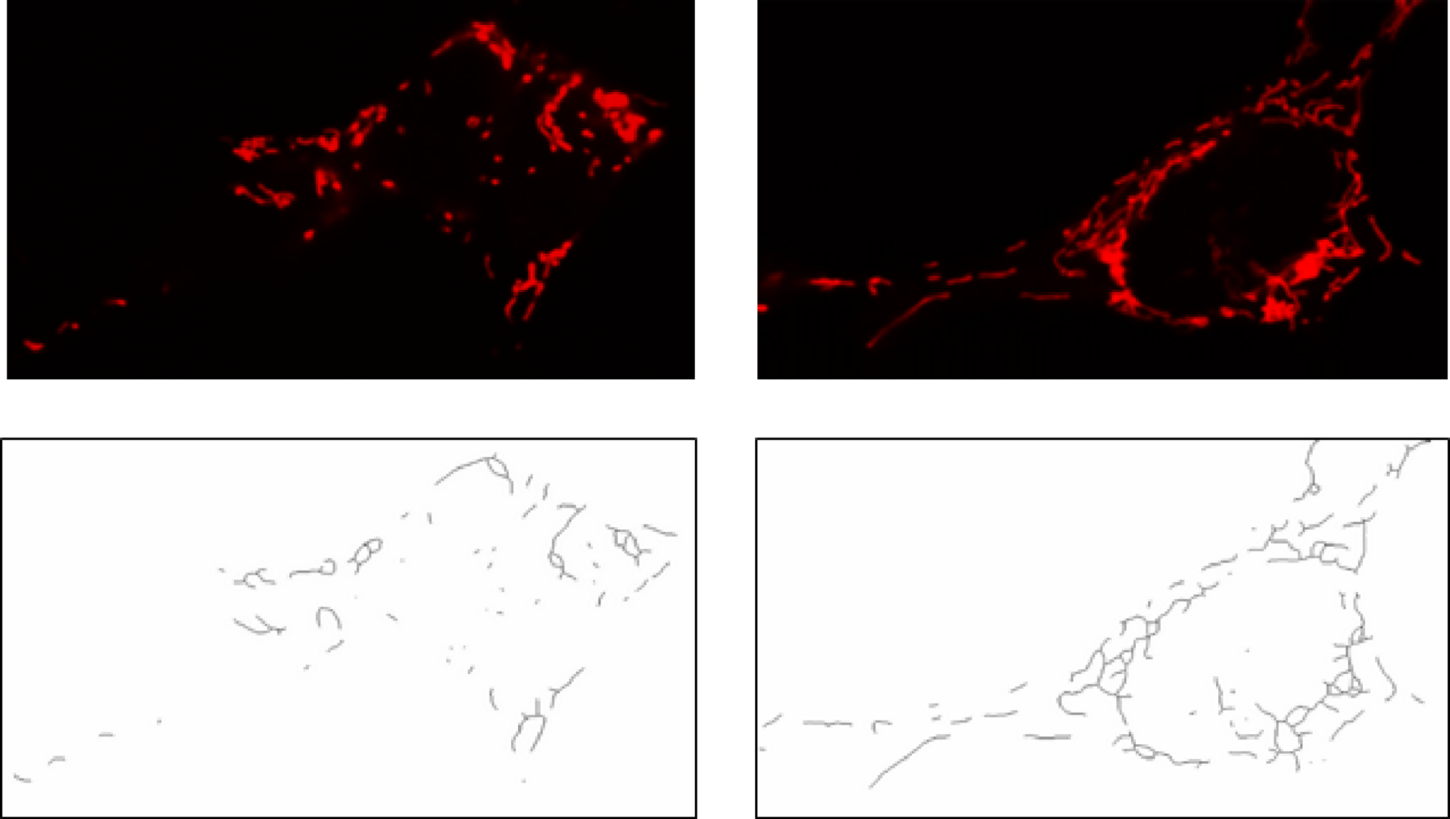
Illustration of mitochondrial network procession by Image J software. Microscopy images of qualitative different mitochondrial network of SH-SY5Y (upper line) and its processing to 2D skeleton (bottom) in order to morphological parametrization, as was used in study

Mitochondrial network morphology was quantitatively assessed using a structured image processing workflow implemented in ImageJ (FIJI), following the method described by Bakare et in 2021 [24]. Fluorescence images acquired by confocal microscopy were first converted into binary 2D representations to enable objective quantification of mitochondrial structures (Fig. 2). Key morphological parameters were extracted to evaluate network integrity and dynamics, including mitochondrial fragmentation, branching, junction density, and average branch length.

Fragmentation was characterized by calculating the ratio of solitary rod-shaped mitochondria to the total number of mitochondrial branches in each cell. Branching was determined by the proportion of junction points relative to the total number of branches. The average branch length was derived by measuring pixel overlap of individual mitochondrial segments under consistent magnification and zoom parameters.

### Cell Counts

Cell counts were evaluated from microscopic images acquired using a light microscope. Images were obtained following the completion of respective treatments. For each experimental group, the average number of cells counted across all acquired images was used as the representative value for a single biological replicate. In total, four independent biological replicates of SH-SY5Y cells were analyzed. This approach allowed for the semi-quantitative assessment of treatment-related effects on overall cell number and viability.

### Statistical Analysis

All experimental data were analysed using parametric statistical methods. Two-way ANOVA was performed to assess the main effects and potential interactions between rotenone and KATP channel modulators across experimental groups. Statistical significance was defined as *p* < 0.05. When a significant main effect of selected factor (KATP modulation and rotenone treatment) was detected, Tukey’s post hoc test was applied for pairwise comparisons. Tukey´s post hoc test results are indicated above each graph (e.g. dzx vs. ctrl) on the right. In cases of a significant interaction between KATP modulation and rotenone, the results of post hoc test are marked with an asterisk above the relevant rows in the graph.

For direct comparisons of specific independent values measured in experimental groups to control cells, Student’s *t*-test was used, with significant differences highlighted by an asterisk above the line connecting the compared groups. The use of t-test is highlighted in legends to respective figure. Additionally, regression analysis was performed to evaluate relationships between variables, assessing both positive and negative correlations. The statistical significance of these correlations was determined using Pearson’s correlation coefficient. The strength and direction of correlations are reported numerically and visualised using colour coding. A significance threshold of *p* < 0.05 was applied throughout.

## Results

The present study aimed to investigate the effects mediated by whole-cell and selective mitochondrial KATP channels. SH-SY5Y neuroblastoma cells were treated for 24 h with two distinct groups of KATP modulators, both under control conditions and under pathological conditions induced by rotenone. For the non-selective modulation of whole-cell KATP channels, pinacidil (opener/agonist) and glibenclamide (blocker/antagonist) were applied. To selectively target mitochondrial KATP channels, diazoxide (opener) and 5-hydroxydecanoate (blocker) were used. The conducted experiments focused on evaluating the general effects of chronic KATP modulation on cell viability, intracellular calcium levels in specific subcellular compartments, and mitochondrial morphology. These outcomes were analysed to assess the cellular responses to KATP modulation under both physiological and Parkinson like pathology conditions.

**Fig. 3:**
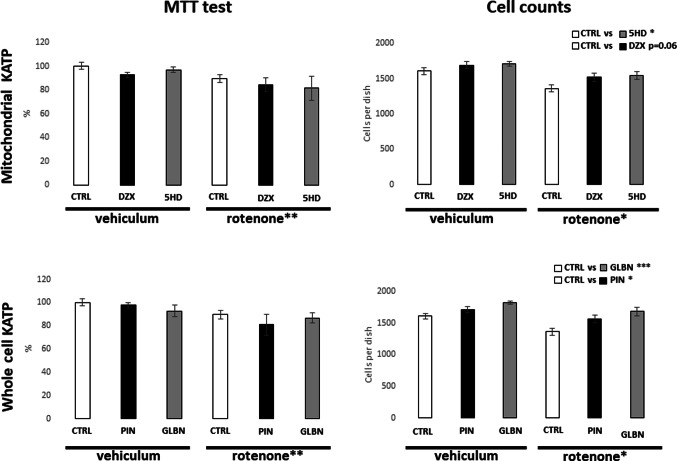
Effect of KATP modulators and rotenone on cell viability and proliferation. Left panel of graphs is showing the results of MTT assay after 24-hour treatment of SH-SY5Y cells with KATP channel modulators under control and rotenone-induced stress conditions. Right panel of bar graphs is showing total SH-SY5Y cell count analysis after same 24-hour treatment. Rotenone treatment significantly reduced cell numbers, as indicated by a two-way ANOVA comparing the three vehicle-treated and three rotenone-treated groups. Significant effects of KATP modulators represent the comparison of combined data from respective KATP treatments under both vehicle and rotenone conditions. Data are presented as means ± SEM; Statistical significance, determined by Tukey’s post hoc test following factorial ANOVA for the relevant factor, is denoted as* for *p* < 0.05, ** for *p* < 0.01 and *** for *p* < 0.001. *veh* vehicle; *rot* rotenone; *ctrl* control; *glbn* glibenclamide; *dzx* diazoxide; *pin* pinacidil; *5HD* 5-hydroxydecanoate)

Cytotoxic effects of the applied treatments were evaluated by MTT assay and the cell density of SH-SY5Y cell cultures. As shown in Fig. 3, a 24-hour exposure to rotenone significantly reduced metabolic activity responsible for MTT turnover to formazan, confirming its cytotoxic effect. In contrast, treatment with either group of KATP modulators alone or in combination with rotenone did not result in any significant changes in MTT turnover compared to their respective controls. Cell count analysis further confirmed the negative impact of rotenone on cell proliferation and viability (Fig. 3). Interestingly, all KATP modulators tested positively affected cell counts indicating an overall proliferating effect of the treatments. However, in the case of diazoxide, the Tukey´s post hoc test revealed only a marginally significant effect. In case of tested couple of antagonists – glibenclamide and 5-hydroxydecanoate – they both exerted a stronger positive influence on cell proliferation than their agonist counterparts. Notably, non-selective modulation of whole-cell KATP channels showed a more pronounced effect compared to the selective mitochondrial KATP modulators.

**Fig. 4:**
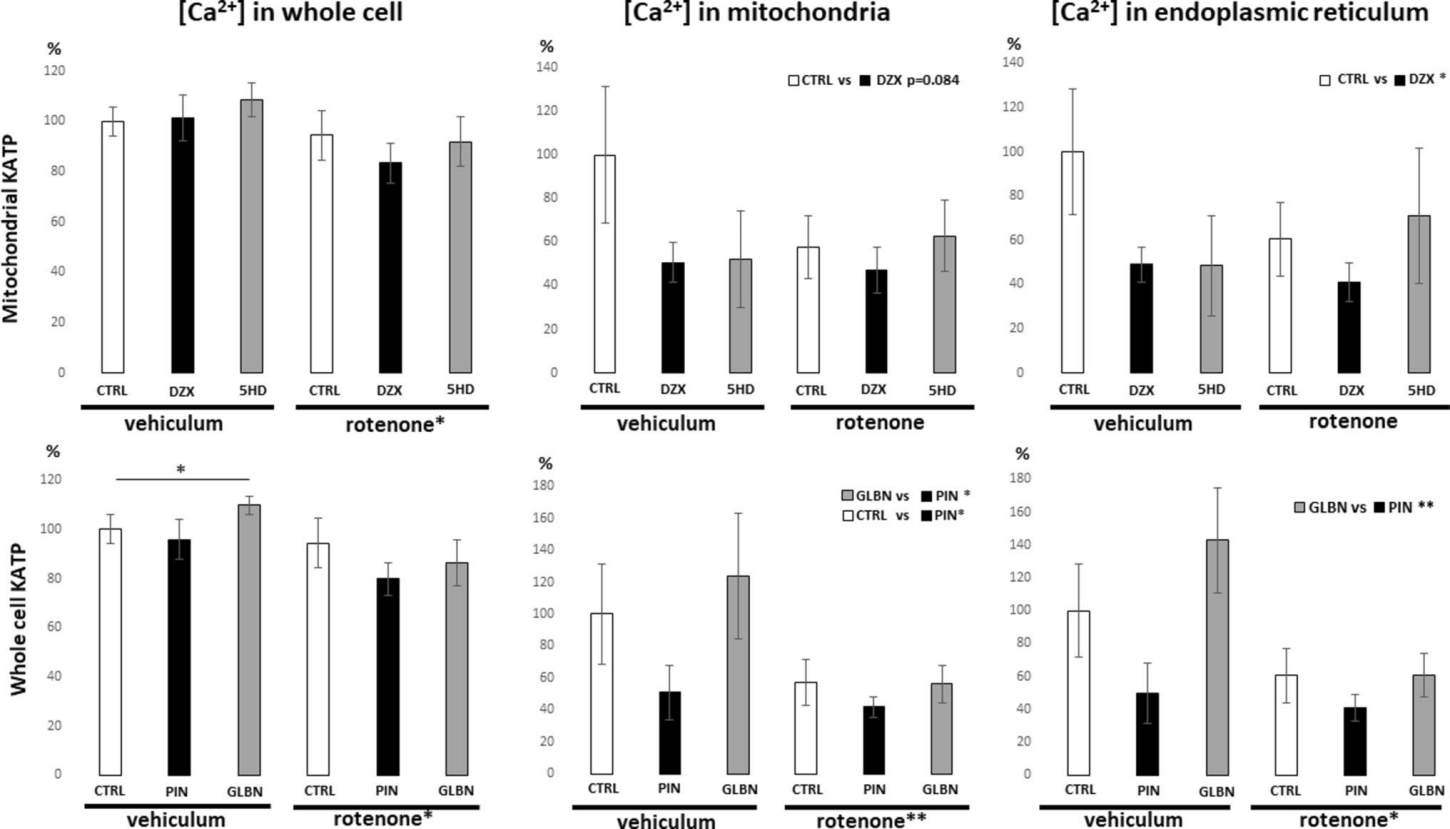
Evaluation of relative intracellular calcium amounts in SH-SY5Y cells. Mean fluorescence intensity of Fluo-4 AM was quantified in the whole cell, mitochondria, and ER compartments after 24-hour treatment with KATP modulators, with or without rotenone. Specific calcium levels in mitochondria and ER were evaluated by colocalization analysis. Significant effects of the respective KATP modulators represent the evaluation of combined data from both vehicle and rotenone conditions, compared to the corresponding combined control/pinacidil or control/glibenclamide groups. Similarly, the effect of rotenone was determined by comparing all rotenone-treated groups with all vehicle-treated groups. Data are presented as mean ± SEM; Statistical significance, determined by Tukey’s post hoc test following factorial ANOVA for relevant factor is denoted as* for *p* < 0.05, ** for *p* < 0.01. Significance revealed by Student´s T test is indicated by * above the compared bars. *veh* vehicle; *rot* rotenone; *ctrl* control; *glbn* glibenclamide; *dzx* diazoxide; *pin* pinacidil; *5HD* 5-hydroxydecanoate)

Further experiments aimed to evaluate the ability of selected treatments to alter intracellular calcium levels, both across the whole cell volume and within specific subcellular compartments. For this purpose, fluorescence microscopy images were analysed post-acquisition. The mean fluorescence intensity of a calcium-sensitive probe (Fluo-4 AM) was measured for the entire cell. Additionally, signal colocalization in acquired images was performed with mitochondrial and endoplasmic reticulum (ER) markers to assess calcium distribution within these organelles (Fig. 4). As shown in Fig. 4, a 24-hour rotenone treatment significantly reduced overall cellular calcium levels. This decrease was also observed within both mitochondria and the ER, as confirmed by two-way ANOVA, particularly in groups treated simultaneously with the non-selective KATP modulators pinacidil and glibenclamide. Although changes in calcium levels were detected in all analysed compartments, the effect was least pronounced at the whole-cell level. A significant increase in calcium signal was observed after 24-hour glibenclamide treatment under control conditions compared to untreated control cells. Among the agonists, both pinacidil and diazoxide induced alterations in mitochondrial and ER calcium homeostasis. However, we can conclude that non-selective KATP opening exerts a more robust effect compared to diazoxide, whose impact on mitochondrial calcium concentration reached only border line statistical significance. When comparing antagonists, glibenclamide exhibited a more intensive effect on intracellular calcium regulation than 5-hydroxydecanoate as well (Fig. 4).

**Fig. 5:**
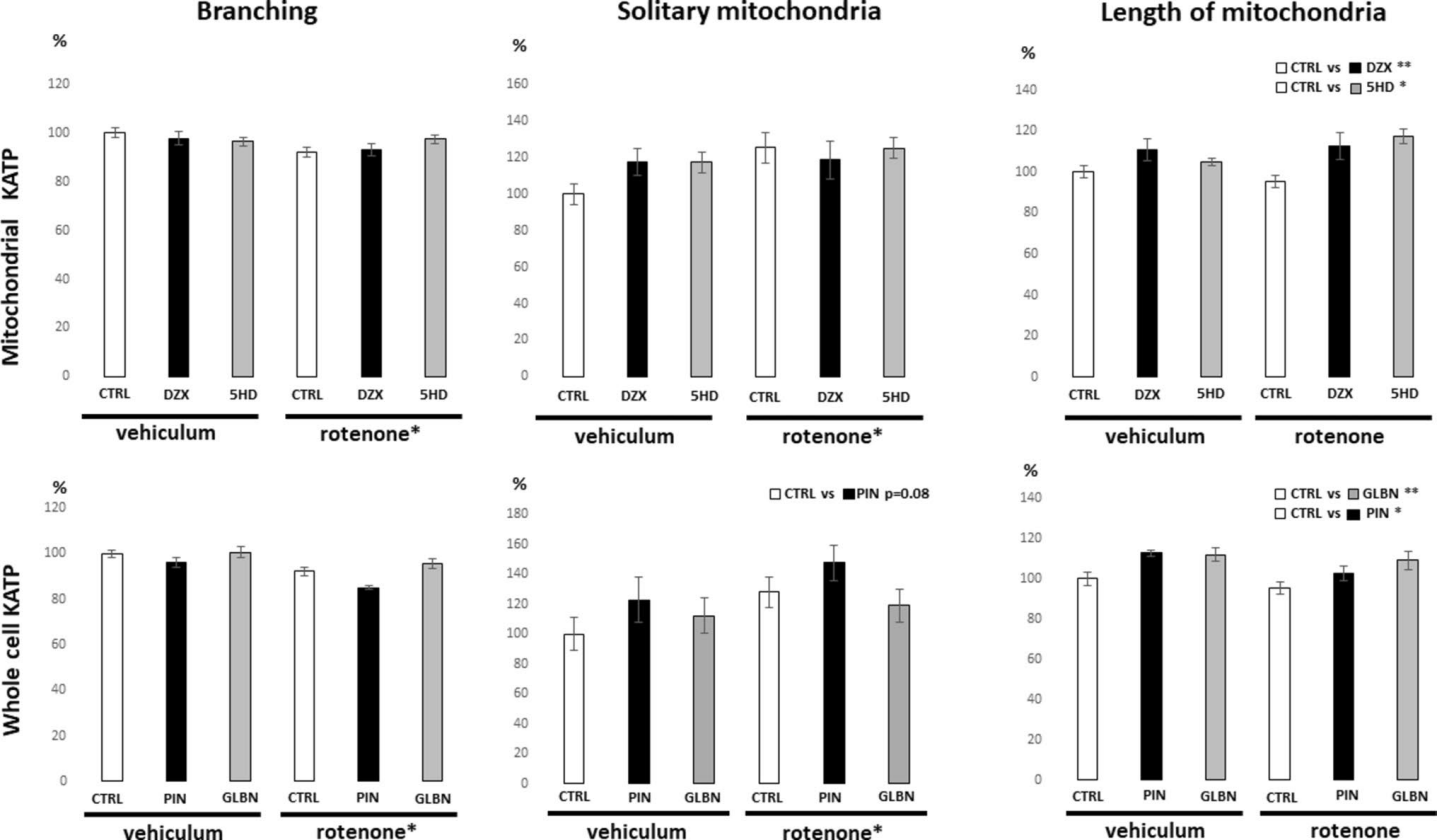
Mitochondrial network morphology under KATP modulation and rotenone stress. Quantitative analysis of mitochondrial morphology after mitochondrial network imaging using MitoTracker Red FM. Rotenone induced significant mitochondrial fragmentation and reduced branching. KATP modulators increased mitochondrial branch length under both control and rotenone-treated conditions. Pinacidil uniquely showed a marginally significant increase in mitochondrial fragmentation. Highlighted significance represents comparisons of combined data from the two experimental groups treated with the respective KATP modulator versus the two control groups. For rotenone, comparisons were made between the three rotenone-treated and three vehicle-treated groups. Data are expressed as means ± SEM; Statistical significance, determined by Tukey’s post hoc test following factorial ANOVA for relevant factor is denoted as* for *p* < 0.05, ** for *p* < 0.01. *veh* vehicle; *veh* rotenone; *ctrl* control; *glbn* glibenclamide; *dzx* diazoxide; *pin* pinacidil; *5HD* 5-hydroxydecanoate)

Fluorescence from the MitoTracker Red FM probe was used to assess mitochondrial network remodelling across the experimental conditions. A 24-hour rotenone treatment led to a significant increase in mitochondrial fragmentation and a concurrent reduction in branching, indicating disrupted mitochondrial dynamics (Fig. 5). None of the KATP modulators tested were able to fully reverse the effects of rotenone on mitochondrial morphology. However, both agonists and antagonists significantly increased the length of mitochondrial branches under control as well as rotenone-treated conditions (Fig. 5). A unique effect was observed with pinacidil, a non-selective opener of whole-cell KATP channels, which resulted in a marginally significant increase in mitochondrial fragmentation (Fig. 5).

**Fig. 6:**
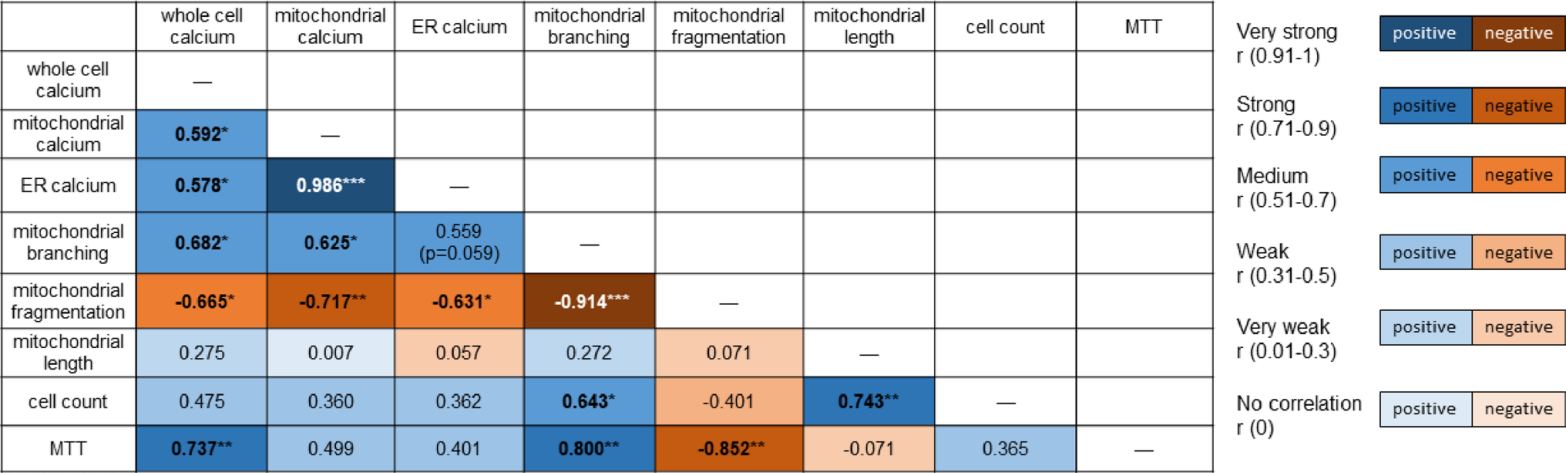
Correlation analysis between mitochondrial morphology, calcium levels, and cell viability. Regression analysis revealed positive correlations between cell count, mitochondrial branch length, and branching. The strength and direction (positive or negative) of each correlation are indicated by color coding, based on the value of Pearson´s correlation coefficient (*r*). Statistical significance, where applicable, is denoted by asterisks

To further explore the relationships between measured parameters, regression analysis was performed within each experimental group. As illustrated in Fig. 6, several significant correlations were detected between calcium concentrations, mitochondrial morphology, and relative cell viability. Cell counts were positively correlated with both mitochondrial branch length and branching frequency. Likewise, mitochondrial branching and whole-cell calcium levels were strongly positively correlated with MTT assay results, suggesting a link between mitochondrial network integrity and cellular viability. In contrast, mitochondrial fragmentation was strongly negatively correlated with MTT assay outcomes.

Mitochondrial network structure was also closely associated with calcium dynamics. Specifically, intra-mitochondrial calcium levels were strongly negatively correlated with mitochondrial fragmentation, while a moderate positive correlation was found between mitochondrial branching and calcium concentration, particularly within mitochondria. A similar trend was also observed for ER-localized and whole-cell calcium levels (Fig. 6).

## Discussion

An intriguing question in neurodegeneration research is how KATP channels, depending on their subcellular localization, differentially regulate calcium homeostasis and mitochondrial architecture. In this study, we compared the effects of mitochondrial versus whole-cell KATP modulation under both physiological and Parkinson’s disease-like conditions. By assessing cell viability, compartmental calcium distribution, and mitochondrial network remodeling after 24 h of treatment, we found that non-selective modulation exerts pronounced effects on both calcium dynamics and mitochondrial morphology, whereas selective mitochondrial modulation produces more subtle, compartment-specific changes. In a case of agonists couple, we identify tendency to conflicting impact on mitochondrial morphology based on their selectivity. Advanced colocalization analysis further revealed a selective decrease in ER and associated mitochondrial calcium. Cytoplasmic levels had higher tendency to remain stable during adaptation, highlighting the nuanced, compartment-specific regulation of calcium by KATP channels. Our findings emphasize the critical role of KATP channel localization in shaping cellular responses and underscore the complex interplay between KATP activity, calcium signaling, and mitochondrial structural integrity.

Cell viability was assessed by measuring MTT reduction to formazan after 24-hour exposure to KATP modulators and rotenone, a process largely dependent on mitochondrial electron transport chain activity and associated oxidoreductases [25]. Therefore, according to expectation, 50nM rotenone with complete inhibitory effect on complex I electron transfer to ubiquinone [26], significantly suppressed MTT reduction (Fig. 3). Phenomenon is consistent with previous reports [27, 28] and was further confirmed also by decrease in cell counts in cultures treated to rotenone. KATP channel modulators did not significantly affect MTT reduction at the tested concentrations, which were selected from the literature as the lowest effective doses [22, 29, 30]. On the other hand, although we did not observe significant changes in metabolic activity after some KATP modulators, the increase in cell counts suggests a possible reduction in cell metabolic activity of experimental cell cultures. This implies that the positive effects on cell counts observed with both KATP antagonists and the non-selective agonist may be associated with a relative decrease in metabolic activity of SH-SY5Y cells. Previous studies have reported variability depending on the methodology and model system used; for example, Yilmaz et al. (2015) found no effect of 10 µM glibenclamide or diazoxide in kidney tubular cells [31], whereas Maqoud et al. (2024) observed a significant negative effect of 100 nM glibenclamide in glioblastoma U87 cells [32]. In this context, our findings obtained with an original treatment protocol contribute valuable additional evidence, with MTT data further supported by direct cell counts.

The most pronounced effects on cell counts were observed with KATP antagonists compared to agonists, with non-selective modulators producing stronger changes than selective mitochondrial ones (Fig. 5). The effects of diazoxide and glibenclamide in this study are consistent with our previous findings in undifferentiated SH-SY5Y cells after 24-hour treatment. Chronic glibenclamide increased cell counts significantly, while diazoxide showed no significant effect. In our earlier work, this rise in viability was further supported by enhanced mitochondrial respiration in glibenclamide-treated cells and longer mitochondrial branches after both treatments [22]. In present study, higher cell counts after 24-hour KATP modulator treatment were accompanied by longer mitochondrial branch lengths (Fig. 6). This supports the idea that KATP channel modulation contributes to maintain mitochondrial network integrity and may play a protective or adaptive role under specific cellular conditions. Interpreting our findings is challenging, as few studies use comparable experimental conditions. Most research with KATP modulators addresses short-term treatments (minutes to hours) and focuses on functional changes such as ion fluxes, membrane potential, or acute mitochondrial responses, whereas data on longer-term effects, especially on cell proliferation or viability, remain scarce. One of the few studies with a comparable approach is that of Núñez et al. (2013), where several days of glibenclamide treatment at a similar concentration inhibited proliferation in a breast cancer cell line through G0/G1 cell cycle arrest. In the same work, the KATP agonist minoxidil exerted the opposite effect by promoting proliferation [33]. In contrast, our present findings demonstrate a proliferative effect of glibenclamide on SH-SY5Y cells. Variability in responses to KATP modulators is common among experimental models, though it is generally accepted that agonists enhance mitochondrial respiration, whereas antagonists exert inhibitory effects [34]. This simplified view does not consider the physiological state of the cells. Riess et al. (2008) showed that the effects of KATP modulators on mitochondrial function depend on the energetic status of the system: under energy deficiency, agonists such as diazoxide and pinacidil stimulate respiration, whereas under sufficient ATP supply they may paradoxically suppress ATP production. This dual behavior highlights the complexity of KATP modulation and the need for contextual interpretation [35]. KATP channels, as cellular energy sensors, appear to mediate rapid adjustments of intracellular metabolism, whereas chronic activation or inhibition can produce divergent and not readily predictable effects on cell fate. To clarify the temporal dynamics and context-dependence of KATP relevance in SH-SY5Y cells, future studies should employ extended time-course experiments across different energetic states and combine cell counts, functional assays, and quantification of KATP expression — an approach that would address a major limitation of the present and other published studies.

Given the established link between KATP channels and calcium regulation, we examined calcium distribution in three compartments - cytoplasm, ER, and mitochondria - to place our findings in the context of calcium homeostasis. Our results indicate that KATP modulators affect calcium content in all three compartments, in line with previous reports [36–38]. A strong correlation between ER and mitochondrial calcium changes (Fig. 6) supports their well-known interplay in calcium buffering and signaling [39], whereas cytoplasmic calcium showed a distinct pattern compared to this tandem response. Statistical analysis of whole-cell calcium levels showed significant effects only in cells treated with glibenclamide or rotenone after 24 h. Glibenclamide induced a mild but significant increase in calcium under control conditions (Student’s t-test), consistent with previous findings that KATP antagonism can elevate intracellular calcium [22]. However, this effect was not confirmed as significant by two-way ANOVA in the present study. In contrast, 50 nM rotenone significantly decreased calcium levels in the whole cell, mitochondria, and ER. While rotenone is often associated with calcium elevation linked to apoptosis induction, such effects are typically reported at much higher concentrations (e.g., 10 µM for 4 h), whereas lower doses appear insufficient to elicit this response [40]. Further compartment-specific analysis revealed a consistent, rotenone-independent decrease in both mitochondrial and ER calcium levels following treatment with KATP channel agonists. Within the mitochondrial network, a more pronounced effect was again observed for pinacidil, the non-selective agonist, whereas the diazoxide-induced calcium decrease reached only marginal statistical significance. This aligns with previous reports of KATP agonist-induced mitochondrial calcium efflux [36]. The decline in ER calcium is expected, although mechanistic studies addressing direct KATP effects at the ER are still lacking. The fact that these subcellular changes are not reflected at the whole-cell level corresponds with our earlier findings, where diazoxide had no effect on total calcium [22]. Altogether, segmentation of microscopic images with advanced colocalization analysis provides detailed insight into subcellular calcium dynamics, revealing a selective decrease in ER and associated mitochondrial calcium while cytoplasmic levels remain stable during 24 h of adaptation to KATP agonists.

Regarding KATP antagonists, glibenclamide again showed greater efficacy than 5-hydroxydecanoate. A significant increase in calcium content was observed in both mitochondria and ER following glibenclamide treatment compared to pinacidil, whereas 5-hydroxydecanoate had no comparable effect relative to diazoxide (Fig. 6). This highlights the higher potency of non-selective KATP modulation in altering subcellular calcium homeostasis. Given the lack of significant effects of glibenclamide in ER and mitochondria compared to controls, these observations likely reflect calcium influx from the extracellular space, with only marginal impact on intracellular stores. A strong correlation between subcellular calcium concentrations, mitochondrial network remodeling, and overall cell viability was evident. Elevated calcium levels – whether in the cytoplasm, mitochondria, or ER – negatively correlated with mitochondrial fragmentation, while in case of mitochondrial branching we observed positive correlation with calcium (Fig. 6). These findings underscore the dose-dependent impact of calcium on mitochondrial physiology.

Chronic calcium overload is generally associated with detrimental effects on mitochondrial physiology [41]. In the context of mitochondrial morphology, excessive calcium typically promotes fission and fragmentation, but such changes become evident only once a threshold of “calcium overload” is surpassed [42]. Tan et al. (2011) reported a similar phenomenon, distinguishing two modes of calcium elevation induced by the ionophore 4Br-A23187 during a brief 30-minute exposure. At higher, pathophysiological calcium levels, mitochondria exhibited pronounced fission and rounding, indicative of cytotoxicity. In contrast, moderate calcium increases caused only subtle reductions in mitochondrial length accompanied by enhanced mitochondrial motility, with these changes persisting for up to 90 min after exposure [43].

Our data further suggest that chronic calcium elevation below the toxic threshold may have protective or stimulatory effects on mitochondrial metabolism and morphology. Conversely, reduced calcium levels appear to promote mitochondrial fragmentation, potentially impairing cellular function (Fig. 6). This may also explain the marginal tendency of the non-selective KATP agonist pinacidil to enhance mitochondrial fragmentation (Fig. 5). The absence of a similar effect with diazoxide is noteworthy, together with its weaker efficacy in reducing mitochondrial calcium concentration, as documented in Fig. 4. This moderate contrast between pinacidil and diazoxide may represent a potential discriminating factor between whole-cell KATP channel activation and selective mitochondrial KATP activation. However, as this remains a working hypothesis, mentioned observation requires further investigation.

**Fig. 7:**
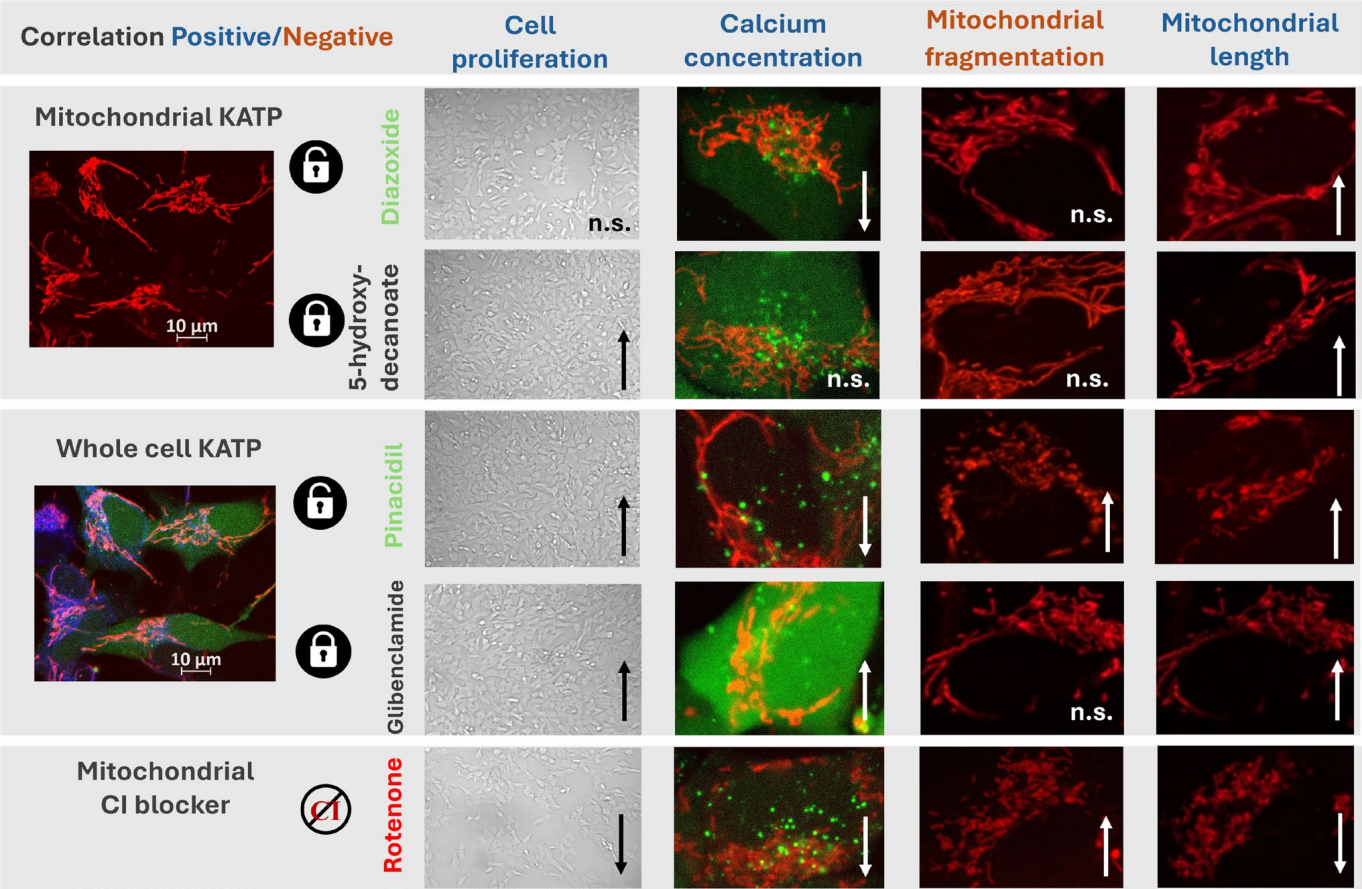
Summary of the obtained results. Recapitulation of the main effects of selected KATP modulators on cell counts, calcium homeostasis, and mitochondrial morphology. The selected images represent the most typical examples of observed changes. The presented images illustrate the documented trends that were identified as statistically significant in the previously shown graphical analyses

**In conclusion**, this comparative study highlights the compartment-specific effects of KATP channels. Distributed across different cellular compartments, KATP channels distinctly influence calcium homeostasis and mitochondrial network remodeling. The observed calcium fluctuations following KATP modulation appear to be not only as secondary phenomena but also as active effectors contributing to mitochondrial structural and functional changes. A schematic summary of the main findings is provided in Fig. 7. Currently available KATP modulators offer selective mitochondrial targeting; however, modulators with restricted selectivity limited to the plasma membrane remain absent from the pharmacological portfolio. Targeting the compartment-specific KATP may represent a promising strategy to enhance beneficial effects while minimizing adverse outcomes in the context of neurodegenerative pathologies.

## Data Availability

The datasets generated during and/or analysed during the current study are available from the corresponding author on reasonable request.
